# Numerical investigation of the haemodynamics in the human fetal umbilical vein/ductus venosus based on the experimental data

**DOI:** 10.1042/BSR20160099

**Published:** 2016-09-29

**Authors:** Taraneh Rezaee, Kamran Hassani

**Affiliations:** * Department of Biomechanics, Science and Research Branch, Islamic Azad University, Tehran, 4515/775, Iran

**Keywords:** abortion, blood flow, 3D computational simulation, Doppler measurement, umbilical vein

## Abstract

Abortion of the fetus due to a disease, in an early stage of pregnancy, has been dramatically increased in the last decades. There is a still lack of knowledge on the various types of diseases which lead fetus to a vulnerable circumstance. The transport of oxygenated blood from the placenta to the human fetus has been an important clinical feature in Doppler velocimetry studies, especially the ductus venosus (DV). The DV connects intra-abdominal portion of the umbilical vein and the inferior vena cava (IVC) at the inlet of the right atrium and is, therefore, important when examining the fetus state of health. An abnormal flow in the DV can indicate a fetal disease such as, chromosomal abnormalities, cardiac defect, hypoxaemia and intrauterine growth restriction (IUGR). The blood flow in the fetal circulation has not been investigated much in detail. The blood flow in the fetal circulation provides necessary information for physician to make a suitable decision on abortion or alternative medical practice before or even after birth. The present study performed a comparative study to quantify the blood velocity in DV by a combination approach based on 3D computational simulation and Doppler measurement. The results showed that the velocity value in DV is significant and can be considered as an indicator of any kind of disease in fetal. The nodal displacement of the model was also analysed. It shows that DV tolerates a higher level of displacement compared with the other regions of the model, whereas the nodal pressure shows different results as the lowest values are located in DV.

## INTRODUCTION

The umbilical cord (UC) is a flexible cord-like structure containing blood vessels and attaching a human or other mammalian fetus to the placenta during gestation. The main role of the UC is to transport nutrient-rich blood and oxygen from the placenta to the fetus and return the blood to the placenta from the fetus. The UC is very important for fetal health and growth. The fetus movements and uterine contractions are not able to occlude the blood flow in umbilical vein due to proper geometry of umbilical [1]. The ductus venosus (DV) originates from the umbilical vein. It sends approximately one-third of the blood flow of the umbilical vein directly to the inferior vena cava (IVC). The fetal liver with its venous vasculature-umbilical, portal veins, DV and hepatic veins and the IVC are the main areas of interest in the investigation of venous blood return to the fetal heart [2]. The investigation shows that the occurrence of pulsations in the umbilical vein during atrial contraction could be an unfavourable indication of a pathological condition [3]. Due to the direct connection between the DV and the umbilical vein, abnormal flow in the DV may also be interesting to be investigated. The characteristics of the blood wave which is propagated by the fetal heart, through the IVC, and enters the DV and the umbilical vein, is an important clinical feature. Abnormal pulsations of this area are associated with numerous diseases such as, chromosomal abnormalities, cardiac defect, hypoxaemia and intrauterine growth restriction (IUGR). An increase in reversed flow during atrial contraction, in the IVC, can show an incorrect cardiac function. In addition, risk of an abnormal flow in DV could be halved if normal flow details were known. As a result, we maybe predict the existence of different congenital heart diseases by studying abnormal flow in DV during the first trimester [4–6]. Congenital heart diseases cause of 20% of spontaneous abortions, 10% of stillbirths and 1% of term pregnancies. Furthermore, cardiac defects are responsible of over 50% of deaths in the children which have been born with a congenital heart disease [5,7]. There are some other investigation for blood flow through ductus venusus such as Pennati et al. [8] who tried to assess a new method to calculate the blood flow rate through the DV in normal human fetuses using available echo-Doppler data. Bellotti et al. [9] used colour Doppler sonography to study umbilical and DV flow in 137 normal fetuses between 20 and 38 week of gestation. He also studied simultaneous measurements of umbilical venous, fetal hepatic and DV blood flow in growth-restricted human fetuses [10]. Kiserud et al. [11] investigated DV shunting in growth-restricted fetuses and the effect of umbilical circulatory compromise.

Toyama et al. [12] did a DV blood flow assessment at 11–14 weeks of gestation and fetal outcome. In the present study, we have presented a computational model to compute the haemodynamic parameters for blood flow in umbilical vein and DV (essential vessels). We have tried to advance the existing models by using more clinical data. The results were compared with some relevant experimental and numerical literature. The presented study is one of the first studies that uses fluid–structure interaction (FSI) to measure the haemodynamic parameters of umbilical vein and DV.

## MATERIALS AND METHODS

### Doppler measurements

**Figure 1:**
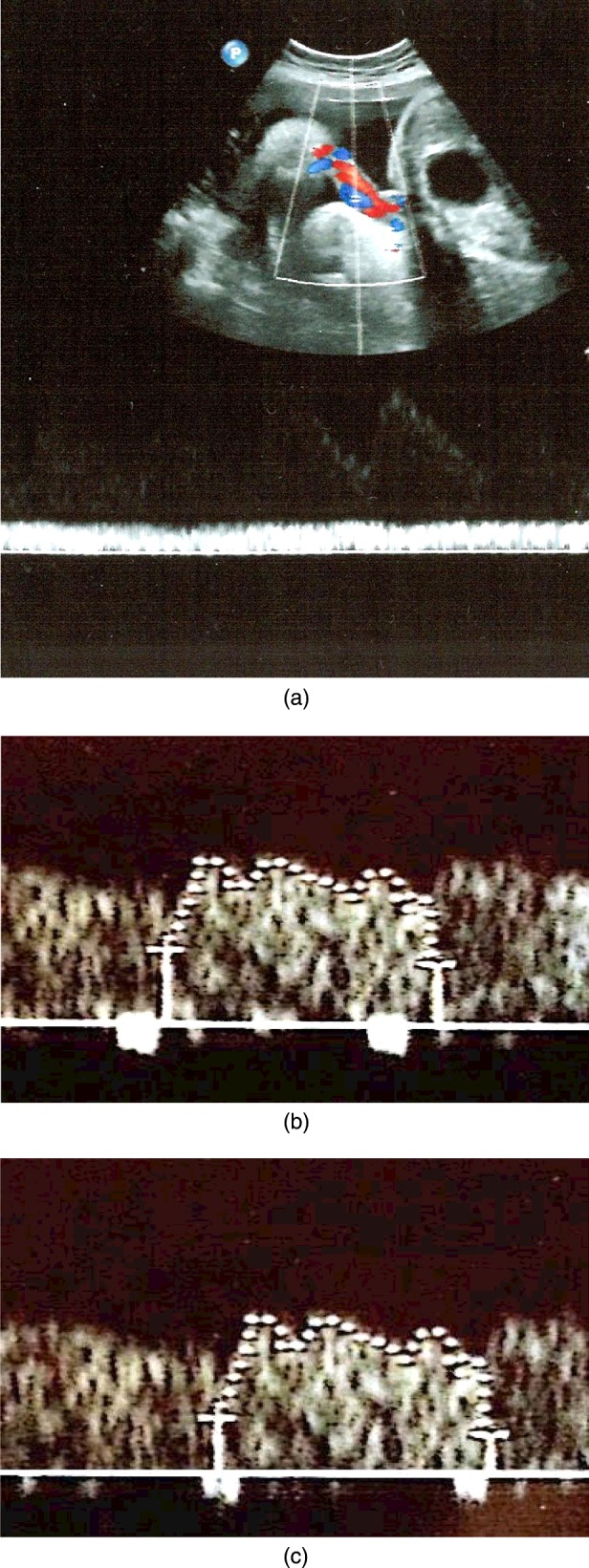
Typical Doppler velocimetric recordings of (**a**) umbilical vein, (**b**) inlet of DV and (**c**) outlet of DV

Eleven healthy pregnant women were investigated in our ultrasound measurements. All had normally grown fetuses with standard biometry (length of femur, head and abdominal circumference). Their pregnancy age was measured by ultrasonography and the average was 34 weeks. All the Doppler examinations were performed by a colour Doppler imaging system (Toshiba Aplio 500 Ultrasound, Toshiba Medical Systems). Since the anatomical characteristics of fetus normally changes during the first months, the obtained data form the geometry of the fetus is not reliable. Therefore, the thickness of veins was measured by Doppler. Furthermore, we used the data of 10 fetuses for our 3D model. In all of the fetuses, Doppler wave forms were recorded both at the isthmus, the outlet portion of the DV and umbilical vein as shown in Figure 1.

### Computational model

The haemodynamic characteristics of blood flow in the human circulatory system are significantly changed by the biomechanical properties of the distensible blood vessel whereas the fluidic force caused by the flow could also alter the nature of the structure [13]. The information regarding Doppler measurements were extracted from the clinical text [14].

In the present study a FSI model was performed to accurately simulate the DV in human fetus under compliant wall boundary based on Doppler imaging data. The governing equations for numerical simulations are consisting of flow equations and coupled FSI ones. The following relations show the constitution and momentum laws [15]:

1 $$\;\frac{\partial u_{i}}{\partial x_{i}}=\;0$$

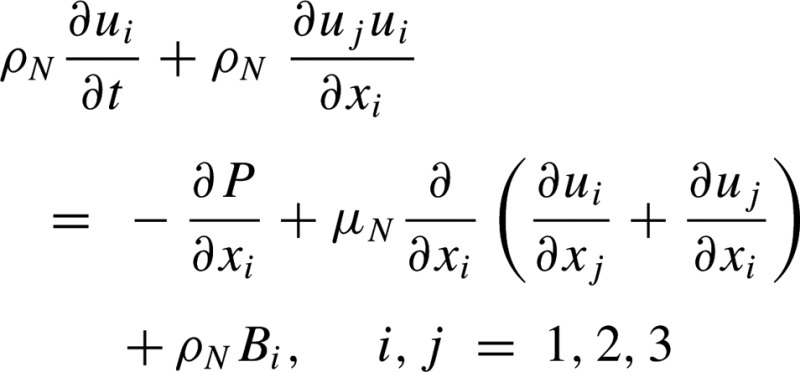


where *I* and *j* denote for the Cartesian directions, *B* is the vector of external forces per mass, *P* refers to the pressure, *u* is the velocity and ρ_*N*_ is the density field of the flow. The above equations are used for to solve the blood flow.

The following boundary conditions were applied to the FSI areas near the walls:

2 $$\mathrm{Kinematics}\;\mathrm{condition}\;\to \;d_{f}\;=\;d_{s}$$

3 $$\mathrm{Dynamics}\;\mathrm{condition}\;\to \;n\cdot \;\sigma_{f}\;=\;n\cdot \;\sigma_{s}$$

where *d*_f_ and *d*_s_ are the displacement of the fluid and solid. σ_f_, σ_s_ represent the fluid and solid stresses and *n* is the normal vector to the interface area [15].

We have used fluid interaction structure methodology developed previously by ourselves [14]. The fluid forces are equivalent to Lagrange multipliers using a non-ideal weak formulation of fluid dynamics. This led to the loading conditions expressed by eqn (4) [15].

4 $${\left(\sigma \cdot n\right)}_{\mathrm{Fluid}}={\left(\sigma \cdot n\right)}_{\mathrm{Solid}}$$

where ***σ*** is the stress tensor and ***n*** is the normal vector to the FSI boundary. Fluid dynamics were solved using the continuity and incompressible Navier–Stokes equations using a full stress tensor [15]. The effect of the compliance of the wall also was quantified with respect to the energy efficiency and haemodynamics of the flow through such connection. On the other hand, the pressure force and wave form incurred by the flow on the vessel wall was investigated, followed by the study how they will change its structure and induce certain disease such as arterial/venous hypertension [16].

### Solid property and modelling

It is appropriate to use thin shell element, defined by two curved surface coordinates, to model such structure. In the theory of shells, the bending and membrane strain are merged in the energy expression, the coupled deformations including the stretching and change of the middle surface curvature are required to predict the stress/strain of the shell element [17,18].

In the present study, 3D-Solid-Quadratic shell elements due to the nature of the problem and some special numerical concerns were used. For QUAD shell element, it gives excellent results for both bending and membrane dominated problem like the coupled structure and blood flow interaction problem [16]. Automatic dynamic incremental nonlinear analysis (ADINA) was used in the present study to solve the structure part of the problem.

### Fluid property and modelling

Computational fluid dynamics (CFD), also from ADINA, was used to solve the fluid part of the problem, with the assumption that the blood flow is homogeneous, incompressible and laminar. However, assigning fluid and structure part in a way to have coupled interaction together is very challenging in FSI analysis. This made us to apply unphysical boundary conditions that lead to severe convergence problems. Initially the fluid domain is unaware of the constraint of the structural domain, and vice versa. If the iteration converges then this discrepancy will be settled, but sometimes the initial phase is so ill posed that convergence is practically impossible to obtain [19].

The artificial compressibility has a natural physical explanation in FSI simulation: the compressibility is defined so that it makes the fluid follow the elastic response of the structure [16].

### Material properties

A single shell layer with a thickness value of 0.5 mm was used globally. The working fluid (blood) was treated as a homogeneous, Newtonian and incompressible with a dynamic viscosity of (*μ*=0.004 kg/m per s), it was also considered as laminar flow and with a no-slip condition at the walls. Material density of (1200 and 1060 kg/m^3^) was assumed for the blood vessel and blood respectively (Table 1). In addition, the Poisson's ratio was considered equal to 0.4999 [20,21].

**Table 1:** Mechanical properties of solid phase (vessel) and fluid phase (blood) in finite element analysis

Material type	Blood	Vessel
Mechanical properties	Homogeneous	Density 1200 kg/m^3^
	Incompressible	
	Newtonian	Young modulus 2.67 MPa
	Laminar	
	Dynamic viscosity μ=0.004 kg/m per s	

### Mechanical properties of vein

In our past experimental tests, we also have measured values for mechanical properties of umbilical vessels by means of clinical approach for some infants, and now we used the results for simulation the haemodynamic of DV in this present investigation. Past studies on umbilical vein stated the mechanical properties of fetal vessels in UC are comparable to those described for vessels after birth. Hence we can use these values in our studies on human fetal analysis during pregnancy [22]. The uniaxial tensile test was performed for the umbilical vein and the mechanical properties of the vein were measured using the test results. The Young's moduli of umbilical vein was considered 2.67 MPa under maximum value of stress [1,2]. However, it should be noted that such linear mechanical properties cannot address the nonlinear mechanical behaviour of most soft tissues, especially umbilical vein. Using nonlinear material models based on strain energy density functions (SEDF) is important for precise simulation and modelling results. In continuum mechanics, SEDFs are used to describe nonlinear mechanical behaviour of hyperelastic materials which are the functions that connect the strain energy density of materials to their deformation gradients. We used the tensile experimental tests results to calibrate the SEDF whom used for axial constitutive modelling. The experimental data were used to fit the incompressible part of the SEDF. Ogden SEDF was used to capture the nonlinear mechanical behaviour of umbilical vein. The Ogden model is carried out to represent the properties of incompressible tissue for finite element analysis of mechanical behaviour of umbilical vein [23]. Ogden material models shows nearly similar behaviour and adequate ability to predict the umbilical vein behaviour for most of the test data range [24]. Hyperelastic material coefficients of umbilical vein under uniaxial loading in Ogden model was considered as α_1_= 3.71, α_2_= −11.89, μ_1_= 3.16, μ_1_= − 2.94 [1,2,25].

### Boundary conditions

The outer surfaces of the structure were assumed to be stress fee and all nodes were fully constrained.

### Geometry acquisition

Pennati et al. [26] examined some normal human fetus of 34 weeks of pregnancy in their investigation on umbilical vein and DV. They evaluated the plane of the branching at the systolic peak for the geometry with a branching angel to 50, constant umbilical vein inlet diameter of 7 mm, umbilical vein outlet diameter of 4.9 mm, DV length, isthmus and outlet diameter 1.5, 3, and 15 mm respectively [26].

So far, the finite element modelling studies assumed the vein wall as a rigid structure. In this investigation an average thickness of vein that was adopted from ultrasonography on human fetus between 33 and 35 weeks of pregnancy, were used for vessel modelling. According to result of Doppler imaging, the thickness of fetus umbilical veins was assumed to be 0.5 mm. We have used Doppler method to evaluate the DV thickness however a previously reported technique (ultrasound measurement) [27] could be used instead. The technique used nuchal translucency (NT) to examine the fetus condition but the fetus anatomical data can be measured using ultrasound: between 11 weeks plus 2 days and 14 weeks plus 1 day of pregnancy (First-trimester). All of our cases were in Third-trimester period and the reported technique could not be used and we were only able to used Doppler method to evaluate the DV thickness. On the other hand, the fetus arterial system is usually examined using Doppler method in Third-trimester and the arterial system is not permitted to be examined in First-trimester by that reported technique. All obtained DV thicknesses which were measured after birth were compared with the Doppler results and we found a very good conformity between our Doppler results to the ones which were measured after birth.

Two 3D models were created based on corresponding vessel diameter for two phases (solid and fluid). Then, they were imported to ADINA for computational analysis.

### Mesh generation

**Figure 2:**
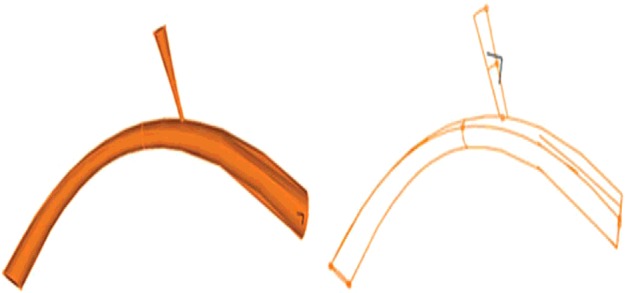
Mesh density analysis is carried out to find the optimum number of elements to get independent results

The mesh generator ADINA CFD and ADINA structure were used to generate the mesh density for the fluidic and structural model respectively [13,28]. An FSI simulation was performed to solve such coupled systems by using staggering partition scheme, a tied CFD and structure solver. The fluid solver CFD starts at first because it is the driving force of the whole problem and provides the structure solver with pressure load, then the solid solver starts and feeds the fluid solver with the displacement of the wall boundary (Table 2). In order to reach to an optimum number of elements, a comparative analysis was out as indicated in Figure 2. In this analysis, the outlet pressure of DV was computed to three different number of elements. Although there is no significant difference between the results of each elements, a higher number of elements were preferred and employed for further analysis.

**Table 2:** The number of elements and modelling parameters

Fluid elements (element group)	(59317) 3D fluid elements	11464 nodes
	Meshing Algorithm: (Delaunay)	
	Nodes per element: 4	
	Mesh type: Free form	
Solid elements (element group)	(16787) 3D solid elements Meshing algorithm: (Delaunay) Nodes per element: 10 Mesh type: Free form	33634 nodes
Number of step: 7000	Constant magnitude: 0.0001	
Maximum number of fluid structure iteration	1000	
Relative force tolerance	1.5	
Relative velocity tolerance	1.5	
Force relaxation factor	0.5	
Displacement relaxation factor	0.5	

### The model

**Figure 3:**
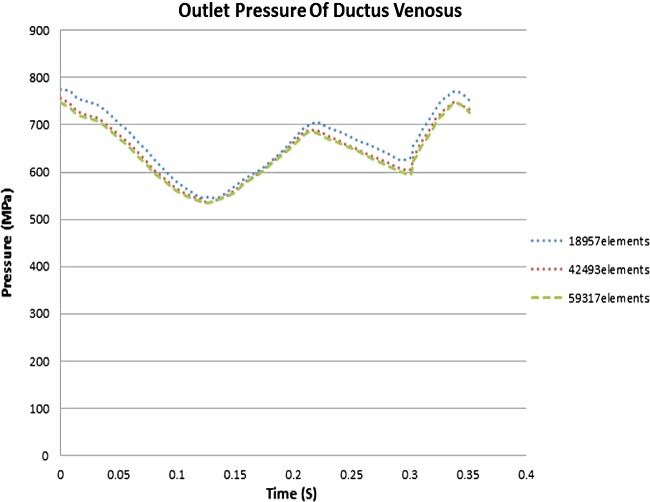
3D model and its connection to the DV

The Ogden material coefficients were computed using nonlinear optimization method and its consistency with experimental data. Thereafter, a DV along with umbilical vein models were established according to the anatomical dimensions of the samples. The model and its connection to the DV are displayed in Figure 3

## RESULTS

**Figure 4:**
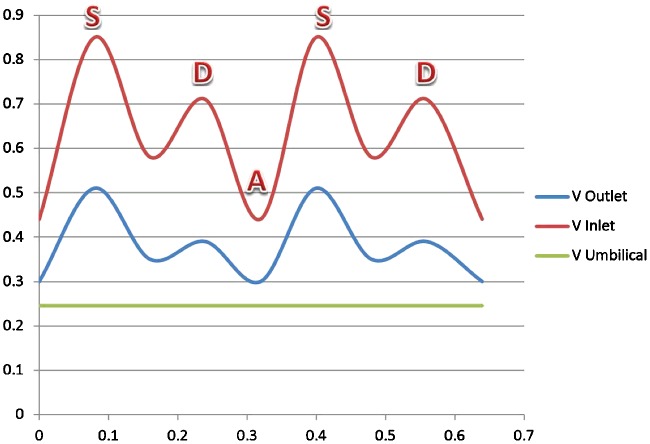
The averaged waves based on 11 normal fetuses between 33 and 35 weeks of gestation during a cardiac cycle in fetal umbilical vein (green line), inlet of DV (red line) and outlet of DV (blue line)

**Figure 5:**
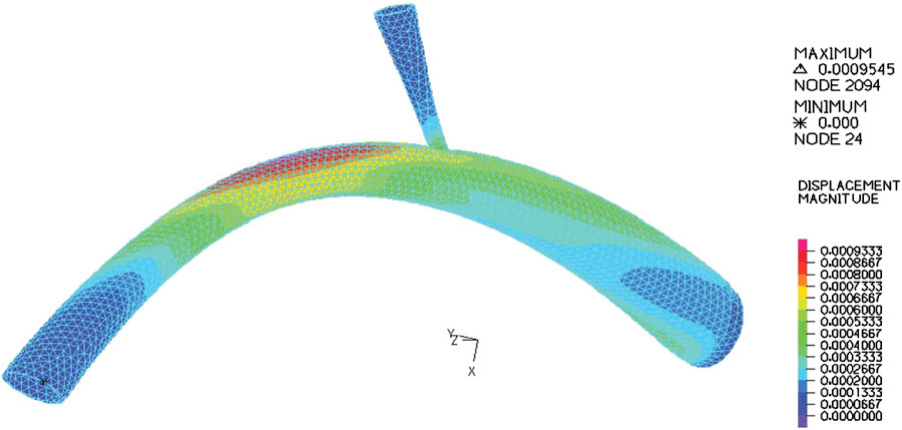
Stress-deformation curve for DV by finite element method

**Figure 6:**
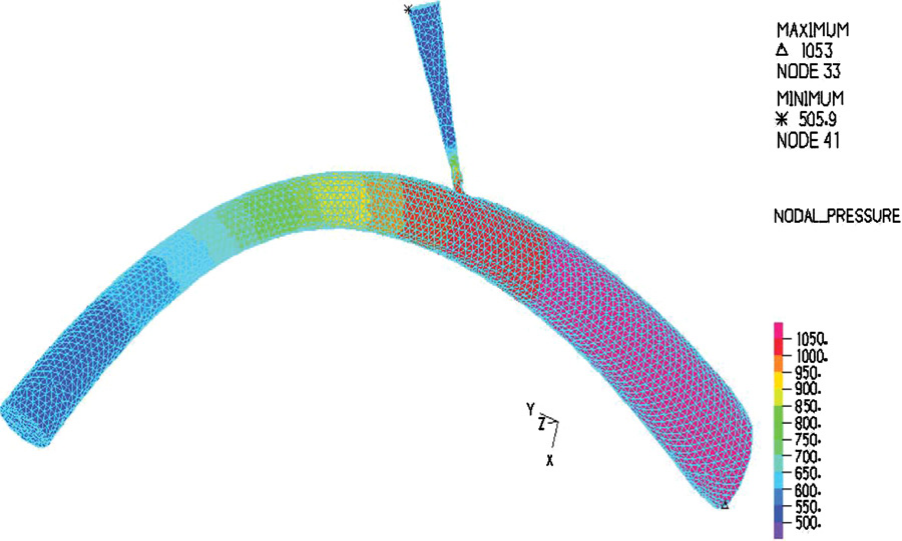
Nodal pressure diagram for DV by finite element method

**Figure 7:**
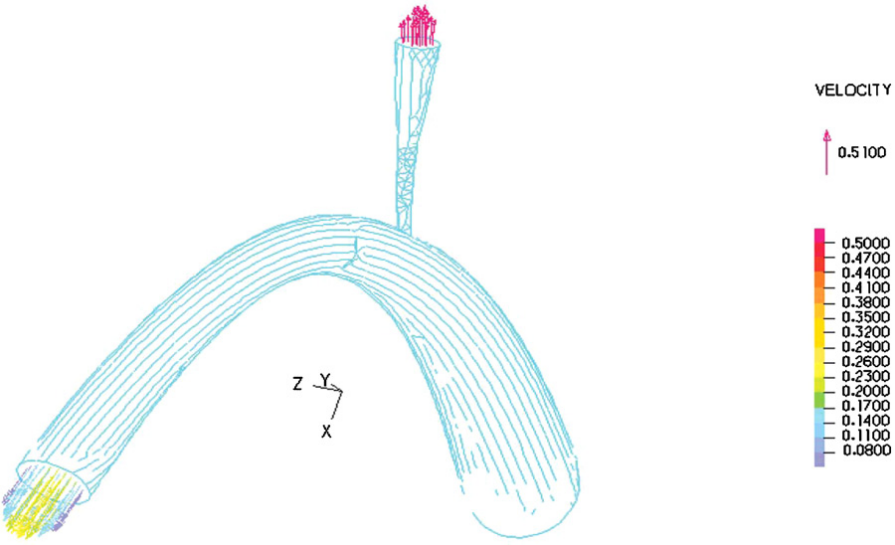
Velocity gradient curve for DV by finite element method

**Figure 8:**
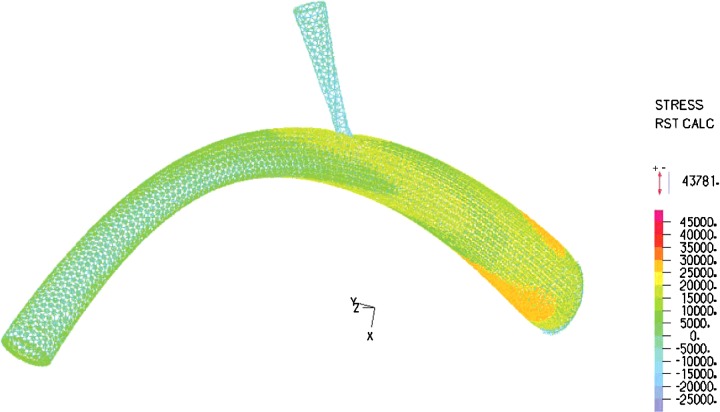
Stress contour of the umbilical vein and DV

In the modelling, blood with constant velocity was inputted to the umbilical vein and the velocity at outlet of DV was computed as presented in Figure 4. From Figure 4, the shape of the blood velocity verifies the modelling results and a simple comparative between the Doppler data and that of numerical results showed good agreement between them [29,30]. A constant value for velocity is observed for umbilical, whereas the inlet and outlet velocity of DV show remarkable fluctuations. This could be related to shape and the mechanical properties of the umbilical wall. The nodal displacement of the model was also analysed in Figure 5 that shows DV tolerates a higher level of displacement compared with the other regions of the model whereas the nodal pressure shows different results as the lowest values are located in the DV (Figure 6). In terms of velocity, the results revealed the velocity of 5.1 cm/s for DV which is significant as this velocity can be considered as an indicator of a disease in this area (Figure 7). Figure 8 displays the stress contour of the umbilical vein and DV.

## DISCUSSION

The understanding of the velocity and pressure induced in the DV would provide a wide range of knowledge about the diseases that can happen for fetus [31]. We have performed a comparative study to assess the blood velocity in the DV by a combination approach based on a 3D computational simulation and Doppler measurement.

A typical umbilical vein was placed between the jaws of the machine and force, induced by a constant strain, was applied to that. The samples were stretched until failure occurred. The uniaxial elastic modulus of the umbilical vein found to be 2.67 MPa which is in good agreement with previously reported results [1,32]. The elastic modulus along with hyperelastic material coefficients of the samples were therefore calculated and implemented into the finite element model. The authors believe that the current information about the velocity and pressure inside DV, due to blood flow, is significant and can provide a suitable knowledge for the surgeons. It has been reported that any alteration in the blood velocity of DV can be considered as a sign of fetus disease [33,34]. The present study numerically investigated the velocity of blood flow in DV and interestingly good agreement between the experimental and numerical results are found. For instance, there is high-velocity jet throughout the lower portion of the ductus as is the low velocity in the umbilical vein [26,35] or the umbilical pressure may show little pulsatility, due to relatively high umbilical compliance [29]. Our numerical results including Figures 6 and 7 were verified by mentioned clinical findings [26,29,35].

In the present study we aimed to calculate the stresses, velocities and the deformations that can be induced during blood flow inside an umbilical vein and DV. The data from the human body was implemented into a finite element model. The results showed that the value of velocity DV is significant and can be considered as an indicator of any kind of disease in fetal. In addition, the nodal displacement of the model was also analysed. It indicates that DV can tolerate a higher level of displacement compared with the other regions of the model, whereas the nodal pressure shows different results as the lowest values are located in DV. It has been reported that any alteration in the blood velocity of DV can be considered as a sign of fetus disease.

## CONCLUSION

The present study numerically investigated the haemodynamics of blood flow in DV and good agreement between the experimental and numerical results were found. It was confirmed that the velocity and pressure change significantly across the ductus venous because of complex geometry. We believe that the presented model is a useful tool for analysing the haemodynamics factors involved in fetus abnormities.

## Abbreviations

ADINA: automatic dynamic incremental nonlinear analysis
CFD: computational fluid dynamics
DV: ductus venosus
FSI: fluid–structure interaction
IVC: inferior vena cava
IUGR: intrauterine growth restriction
SEDF: strain energy density functions
UC: umbilical cord
